# The expression and clinical significance of CFAP65 in colon cancer

**DOI:** 10.1186/s12876-024-03317-5

**Published:** 2024-07-11

**Authors:** Yunze Li, Dongmei Ran, Shiva Basnet, Buzhe Zhang, Hongjing Pei, Chenchen Dan, Zixuan Zhang, Liang Zhang, Tianyu Lu, Yifan Peng, Changzheng Du

**Affiliations:** 1 Key Laboratory of Carcinogenesis and Translational Research (Ministry of Education), Department of Unit III & Ostomy Service, Gastrointestinal Cancer Center, Peking University Cancer Hospital & Institute, 52 Fucheng Road, Beijing, 100142 P.R. China; 2 Department of Pathology, Southern University of Science and Technology Hospital, Shenzhen, 518055 Guangdong China; 3 Digestive Tumor Center, Southern University of Science and Technology Hospital, Shenzhen, 518055 Guangdong China; 4 Department of Gastroenterology, Southern University of Science and Technology Hospital, Shenzhen, 518055 Guangdong China; 5 Department of Unit III & Ostomy Service, Gastrointestinal Cancer Center, Peking University Cancer Hospital & Institute, Beijing, 100142 P.R. China; 6 Beijing Tsinghua Changgung Hospital & Tsinghua University School of Medicine, 168 Litang Road, Changping District, Beijing, 102218 P.R. China

**Keywords:** CFAP65, Colon cancer, Prognosis, Pathology, Surgery

## Abstract

**Background:**

CFAP65 (cilia and flagella associated protein 65) is a fundamental protein in the development and formation of ciliated flagella, but few studies have focused on its role in cancer. This study aimed to investigate the prognostic significance of CFAP65 in colon cancer.

**Methods:**

The functionally enriched genes related to CFAP65 were analyzed through the Gene Ontology (GO) database. Subsequently, CFAP65 expression levels in colon cancer were evaluated by reverse transcription and quantitative polymerase chain reaction (RT-qPCR) and immunoblotting in 20 pairs of frozen samples, including tumors and their matched paratumor tissue. Furthermore, protein expression of CFAP65 in 189 colon cancer patients were assessed via immunohistochemical staining. The correlations between CFAP65 expression and clinical features as well as long-term survival were statistically analyzed.

**Results:**

CFAP65-related genes are significantly enriched on cellular processes of cell motility, ion channels, and GTPase-associated signaling. The expression of CFAP65 was significantly higher in colon cancer tissue compared to paratumor tissue. The proportion of high expression and low expression of CFAP65 in the clinical samples of colon cancer were 61.9% and 38.1%, respectively, and its expression level was not associated with the clinical parameters including gender, age, tumor location, histological differentiation, tumor stage, vascular invasion and mismatch repair deficiency. The five-year disease-free survival rate of the patients with CFAP65 low expression tumors was significantly lower than that those with high expression tumors (56.9% vs. 72.6%, *P* = 0.03), but the overall survival rate has no significant difference (69% vs. 78.6%, *P* = 0.171). The cox hazard regression analysis model showed that CFAP65 expression, tumor stage and tumor location were independent prognostic factors.

**Conclusions:**

In conclusion, we demonstrate CFAP65 is a potential predictive marker for tumor progression in colon cancer.

**Supplementary Information:**

The online version contains supplementary material available at 10.1186/s12876-024-03317-5.

## Background

Colon cancer (CC) is the third most common cancer and the second leading cause of cancer death in the world, with increasing morbidity and mortality among young people over the last decade [1]. Therapeutic outcomes of CC remain to be improved, especially for late-stage tumors [2]. Due to the wide heterogeneity of CC, it is crucial to accurately assess the clinical prognosis after surgery. Therefore, the development of new prognostic biomarkers to stratify patients at different risk of progression is essential to improve the treatment [2].

In order to explore more potential prognostic markers for colon cancer, we used the UALCAN [3] database to obtain a list of genes that may affect the prognosis of colon cancer in the TCGA colon cancer dataset (Supplementary Table 1). Furthermore, we found that CFAP65 (also known as CCDC108) is a strong prognostic factor affecting the overall survival and the disease-free survival of colon cancer patients, among a range of potential prognostic markers. Therefore, we chose CFAP65 as the focus of our subsequent analysis.

CFAP65 is a protein involved in flagella formation and sperm motility which belongs to the cilia and flagella-associated protein (CFAP) family. CFAP65 is needed for basal body migration or docking to the plasma membrane and apical enrichment of F-actin during multiciliogenesis [4]. CFAP65 is a protein with a transmembrane domain and has a strong expression at the equatorial zone, it serves as a scaffold protein on the nuclear surface related to both the acrosome and manchette during spermiogenesis. Correct localization of CFAP65 is essential for the recruitment and transport of manchette-related complex and the acrosome anchoring-related complex [5, 6]. The majority of studies on CFAP65 focus on its role in male sterility. Biallelic mutations in CFAP65 result in acrosomal agenesis as well as multiple morphological abnormalities of the sperm flagellum phenotype, eventually leading to severe asthenospermia [7–9]. In the cancer development, CFAP65 has been demonstrated to participate in mitochondrial retrograde signaling pathway mediated by TFAM depletion, which has been verified to reduce cell proliferation in esophageal, arsenical skin, and prostate cancers, thereby affecting tumor cell proliferation and differentiation [10]. However, the clinical significance of CFAP65 in CC is still unclear. Here, we investigate the expression of CFAP65 in CC and reveal its prognostic significance in the clinic.

## Methods

### Patients

From January 2004 to December 2013, 189 consecutive patients with primary colon adenocarcinoma undergoing radical surgery were enrolled. Patients diagnosed with familial adenomatous polyposis or with clinical criteria for hereditary nonpolyposis colon cancer were excluded from this study. Adjuvant chemotherapy based on and fluorouracil or oxaliplatin is recommended for Stage III tumors and for stage II tumors at high risk for recurrence, such as cancer perforation, pT4N0 with vascular embolization, and/or intestinal obstruction [11]. Chemotherapy was given to the 149 patients with stage III, and to the 29 patients with stage II at high risk. Patients were followed up every three months for the first three years after surgery, every six months for the next two years, and annually after five years. Tumor progression was assessed by serum carcinoembryonic antigen levels, colonoscopy, chest radiography, and computed tomography. Patients who failed follow-up were excluded. All patients signed informed consent prior to treatment, and the study was approved by the ethics committee of Peking University Cancer Hospital (Resolution#: 20,110,225).

### Immunohistochemistry and tissue microarray

The expression of CFAP65 was evaluated by immunohistochemical staining of tumor and adjacent tissues. Immunohistochemistry (IHC) was performed on formalin fixed paraffin embedded tissue with CFAP65 specific antibodies (CCDC108 Polyclonal Antibody, Invitogen, Cat#PA5-113046) on tumor and paracancerous tissues. The tissue microarrays with a 3-mm punch taken from each paraffin-embedded block were used [12]. Immunostaining was performed using the Leica Bond MAX automated Immunostainer (Leica Microsystems, Wetzlar, Germany), with the antibody dilution ratio of 1:500 (v/v). The expression level of CFAP65 was evaluated based on the commonly used immunoreactive score (IRS) system [13], namely, the intensity of immunostaining is divided into 0–3 grades: 0, negative; 1, weak; 2, medium; 3, strong; while the percentage of positive cells scored from 0 to 4: 0, < 5%; 1, 5-25%; 2, 25-50%; 3, 50-75%; 4, > 75%; and the final score is calculated as the intensity multiplied by the score. All samples were classified to high-expression and low-expression, based on a threshold score of 6 (high expression > 6, low expression ≤ 6). The negative control is the staining of slides without primary antibody incubation. All tissues come from enrolled patients. All samples were evaluated by two experienced pathologists who were unaware of clinical information and research results.

### Immunoblotting assay

Total protein was extracted from frozen tissues using lysis buffer as previously described [14]. Proteins were then separated on 10% SDS-PAGE gels and transferred onto nitrocellulose (NC) membranes (Amershan protran, Cat#10,600,002), which were incubated overnight with CFAP65 (CCDC108 Polyclonal Antibody, Invitrogen, Cat#PA5-113046) and β-actin (Anti-beta actin, ZSGB-BIO, Cat#TA-09) specific antibodies at 4℃. Blots were probed with horseradish peroxidase-conjugated secondary antibodies (ZSGB-BIO, Cat#ZB-2301, ZB-2305), followed by detection with enhanced chemiluminescence (ECL, Advansta, Cat#230329-20) by ChampChemi 610 Plus System (SAGE, China).

### RNA extraction, reverse transcription and quantitative real-time polymerase chain reaction (RT-qPCR)

Total RNA was isolated from ground tissue using a total RNA isolation kit (Vazyme, Cat#RC112-01). Complementary DNA (cDNA) was reverse transcribed from 2 µg of isolated total RNA using a reverse transcription kit (Vazyme, Cat#R333). The reverse transcription PCR program was 50℃ for 15 min and 85℃ for 5 s. cDNA was diluted 10 times with RNase-free water as a template for qPCR. Quantitative real-time PCR (qPCR) was performed on a QuantStudio 7 Flex system (Thermo fisher, USA) with a SYBR Green PCR Kit (TransGen, Cat#AQ132-21). B2M served as an internal control for the normalization of CFAP65 expression. The primers used are: B2M (F: AGGCTATCCAGCGTACTCCA; R:CTGCTTACATGTCTCGATCCCA); CFAP65 (F: ATCCCTGCCATCAACGACAG; R: CAGATGACCCTCTTGTTCACTC).

### Statistical analysis

The collection and analysis of clinical data was performed using SPSS 24.0 package (IBM Corp., Armonk, NY). Paired t-test to evaluate the differential expression of CFAP65 between tumor tissues and normal tissues. The relationship between the expression level of CFAP65 protein and the clinicopathological characteristics of patients was evaluated using a chi-square test. Disease-free survival (DFS) and Overall survival (OS) were evaluated using the Kaplan-Meier method, and differences between the curves were tested using log-rank test. Cox proportional hazard regression models (Enter method) are used to determine independent prognostic factors which affects disease-free survival time. All tests were bilateral, and *P* < 0.05 was considered statistically significant.

### GO enrichment analysis

The UALCAN cancer database was used to search for genes associated with CFAP65, and the gene list was used for enrichment analysis. The clusterProfiler package in R language was used for GO enrichment analysis, and the ggplot2 package was used for plotting [15]. Data with *P*-value < 0.05 in the enrichment results were considered statistically significant.

## Results

### GO enrichment analysis of genes and biological function associated with CFAP65

**Fig. 1:**
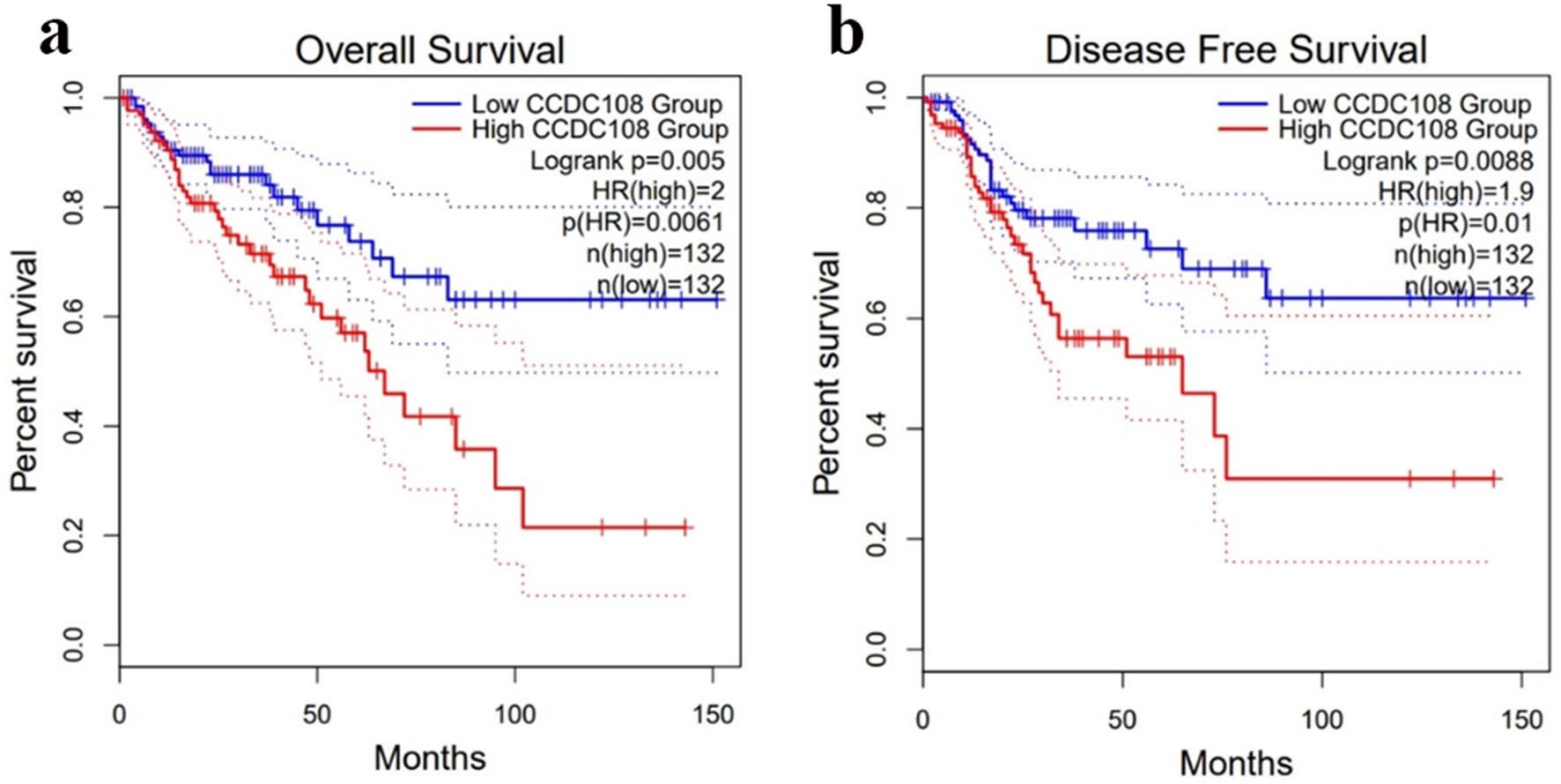
(**a**). The effect of CFAP65 expression level on the overall survival of colon cancer. (**b**). The effect of CFAP65 expression level on the disease-free survival of colon cancer

**Fig. 2:**
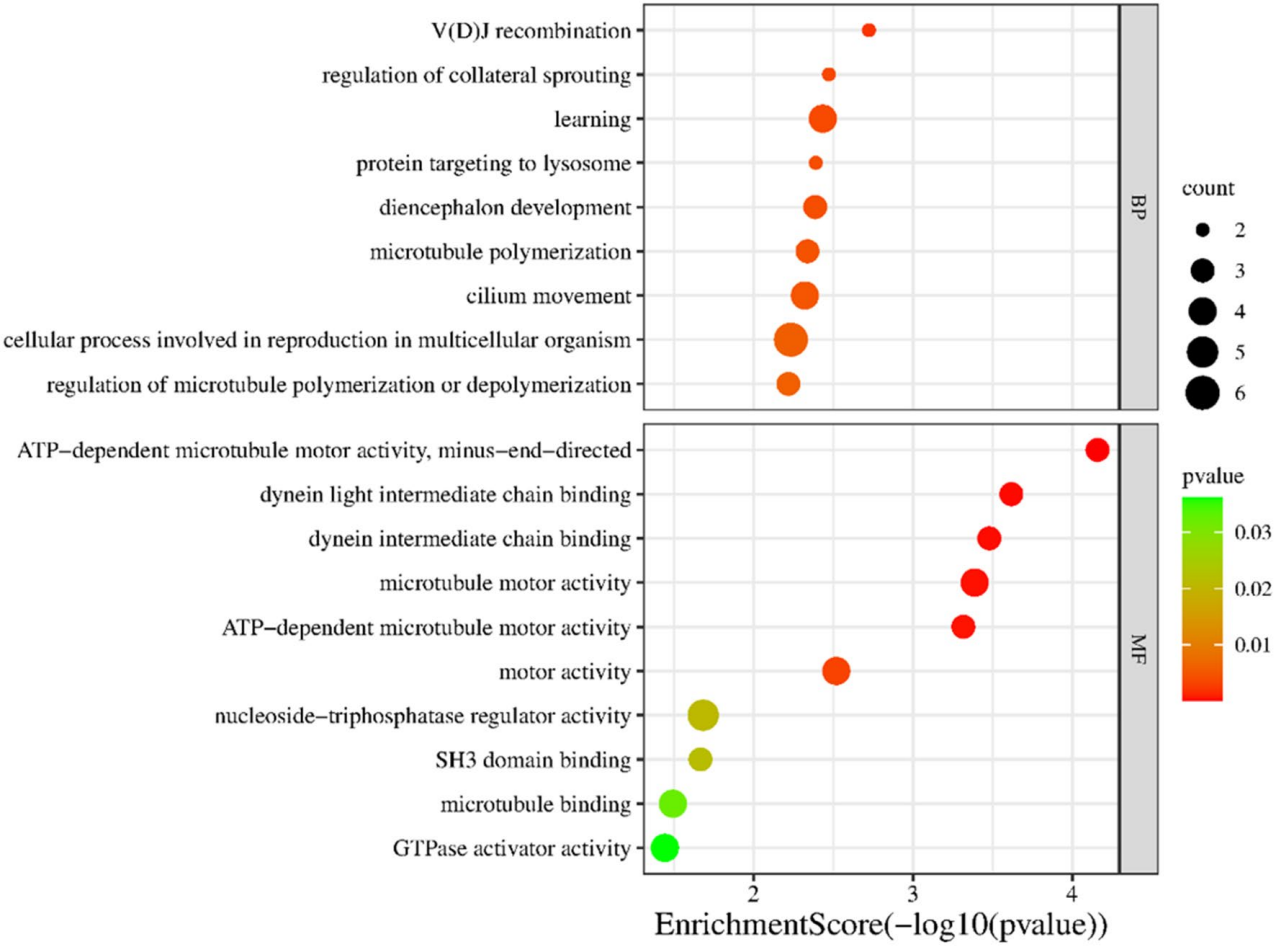
GO enrichment analysis of CFAP65 associated genes in colon cancer

We screened a series of potential prognostic factors for colon cancer through the UALCAN database and found that CFAP65 is a strong prognostic factor because patients with high expression of CFAP65 have significantly poorer prognosis in both overall survival and disease-free survival compared to patients with low expression of CFAP65 according to the TCGA database (Fig. 1). Then we obtain a total of 135 genes are associated with CFAP65 in colon cancer by searching the UALCAN cancer database, and these genes were analyzed for GO enrichment using the clusterProfiler package. The enrichment of CFAP65-related gene sets in terms of molecular function (MF) and biological process (BP) is shown in the Fig. 2. In terms of molecular functions and biological processes, genes were mainly concentrated in items related to cell motor activity and signal transduction, suggesting that CFAP65 may play an important role in cell motility potentially associated with metastasis of colon cancer.

### The expression of CFAP65 and its association with clinicopathological parameters

**Fig. 3:**
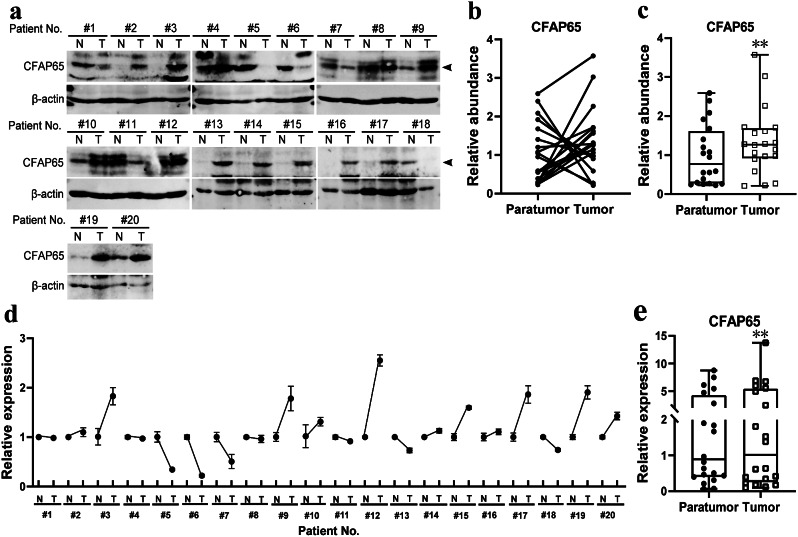
The expression of CFAP65 in tumor and paratumor tissue in colon cancer. (**a**). Immunoblotting of CFAP65 in tumor and paratumor tissue (*n* = 20); arrows show the bands of CFAP65 protein; (**b**). Quantification of CFAP65 for each pair of samples; (**c**). Box plot of CFAP65 protein abundance between tumor and normal tissues; (**d**). Relative mRNA expression of CFAP65 for each pair of samples, determined by RT-qPCR assay; (**e**). Box plot of CFAP65 mRNA expression between tumor tissues and normal tissues. N, normal (paratumor) tissue; T, tumor tissue; **, *P* < 0.01

**Fig. 4:**
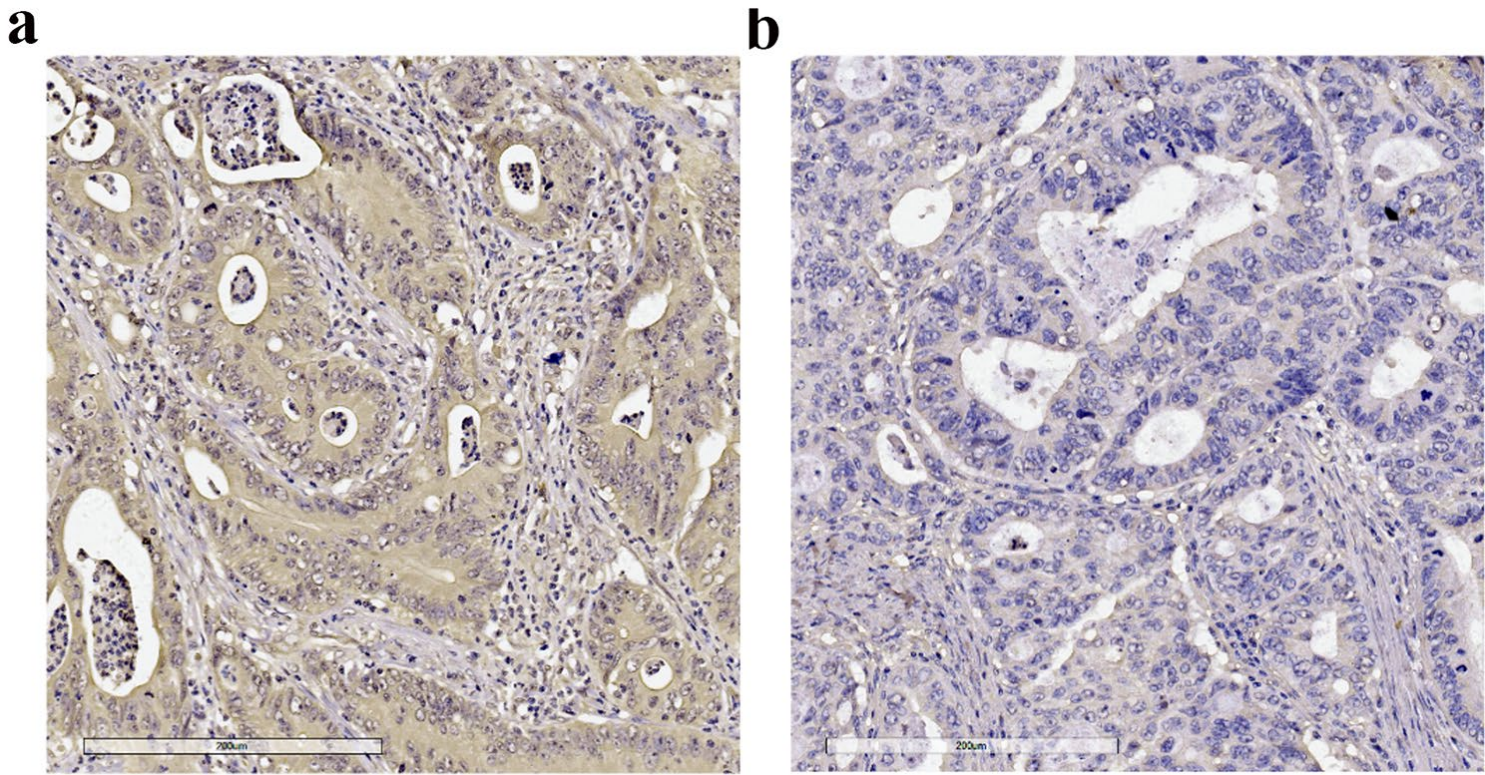
The immunohistochemical staining of CFAP65 expression in colon cancer tissue. (**a**). High-expression; (**b**). Low-expression; Magnification: 200x

**Fig. 5:**
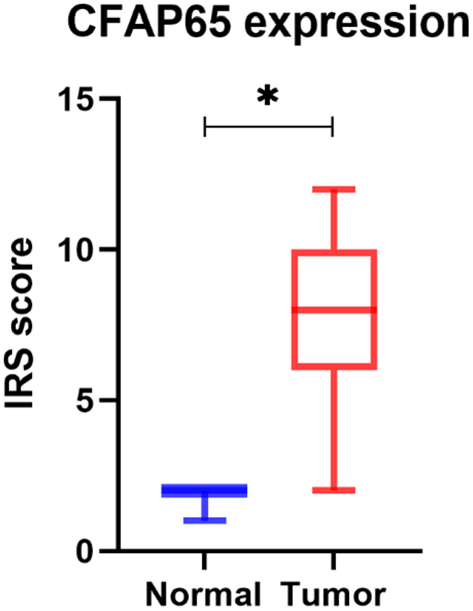
The box plot of CFAP65 expression in tumor and normal tissues. *:*P* < 0.05

To determine the expression level of CFAP65 in colon cancer and normal tissue, we detected CFAP65 expression in 20 pairs of frozen samples using both immunoblotting (Fig. 3a-c) and RT-qPCR (Fig. 3d-e), finding that CFAP65 displayed a higher expression in tumors compared to their matched paratumor tissue (Fig. 3c, e). To identify the clinical significance of CFAP65 in colon cancer, totally 189 patients were recruited retrospectively, with a median age of 69. The clinicopathological characteristics of the patients are listed in Table 1. By immunohistochemical staining, CFAP65 was observed in the cytoplasm, as shown in Fig. 4. According to the immunostaining score, the expression of CFAP65 was evaluated in normal and tumor tissues of patients, as shown in Fig. 5. The expression of CFAP65 in colon cancer tissue was significantly higher than that in normal tissue, which is consistent with the TCGA data and the lab work result. To illuminate the relationship between CFAP65 and pathological stage, we compared the expression level of CFAP65 among different stages, finding that there was no significant difference, as shown in Supplementary Fig. 1. The high-expression and low-expression rates in colon cancer were 61.9% (117/189) and 38.1% (72/189), respectively. There was no correlation between the CFAP65 expression and clinicopathologic parameters including age, gender, tumor location, histological differentiation, tumor stage, vascular invasion, and mismatch repair deficiency (Table 2).

**Table 1:** Clinicopathological characteristics of the patients in this study

Clinicopathological parameters	No. (%)
Median age years (range)	69 (35–93)
Sex (M: F)	102: 87
**Location**	
Right colon	76 (40.2)
Transverse colon	16 (8.5)
Left colon	97 (51.3)
**Histological differentiation**	
Well	18 (9.5)
Moderate	154 (81.5)
Poor	8 (4.2)
Mucinous and signet	9 (4.8)
**TNM stage**	
I	14 (7.4)
II	79 (41.8)
III	96 (50.8)
**Mismatch repair deficiency**	
Yes	18 (9.5)
No	171 (90.5)
**Pretreatment serum CEA** **(Mean ± SD**,** ng/mL)**	14.86 ± 38.23
**Lympho-vascular invasion**	39 (20.9) *

*Data on 187 patients were available.

CEA: carcinoembryonic antigen; F: female; M: male; TNM: tumor node metastasis

**Table 2:** The association between the expression of CFAP65 and clinical parameters

Characteristics	CFAP65		*P* value
	High (%)	Low (%)	
	*n* = 117	*n* = 72	
**Gender**			
Male	60 (51.3)	42 (58.3)	0.37
Female	57 (48.7)	30 (41.7)	
**Age (yr)**			
<65	43 (36.8)	31 (43.1)	0.444
≥ 65	74 (63.2)	41 (56.9)	
**Tumor location**			
Right colon	47 (40.2)	29 (40.3)	0.999
Transverse colon	10 (8.5)	6 (8.3)	
Left colon	60 (51.3)	37 (51.4)	
**Histological differentiation**			
Well	8 (6.8)	10 (13.9)	0.217
Moderate	99 (84.6)	55 (76.4)	
Poor	6 (5.1)	2 (2.8)	
Mucinous and signet	4 (3.4)	5 (6.9)	
**T stage**			
T1-2	13 (11.1)	4 (5.6)	0.521
T3	89 (76.1)	56 (77.7)	
T4	15 (12.8)	12 (16.7)	
**N stage**			
N0	60 (51.3)	33 (45.8)	0.467
N1	35 (29.9)	20 (27.8)	
N2	22 (18.8)	19 (26.4)	
Lympho-vascular invasion*			
Yes	22 (19.1)	17 (23.6)	0.466
No	93 (80.9)	55 (76.4)	
**Mismatch repair deficiency**			
Yes	13 (11.1)	5 (6.9)	0.447
No	104 (88.9)	67 (93.1)	

*Data on 187 patients were available

### Association between CFAP65 expression and 5-year survival in colon cancer

**Fig. 6:**
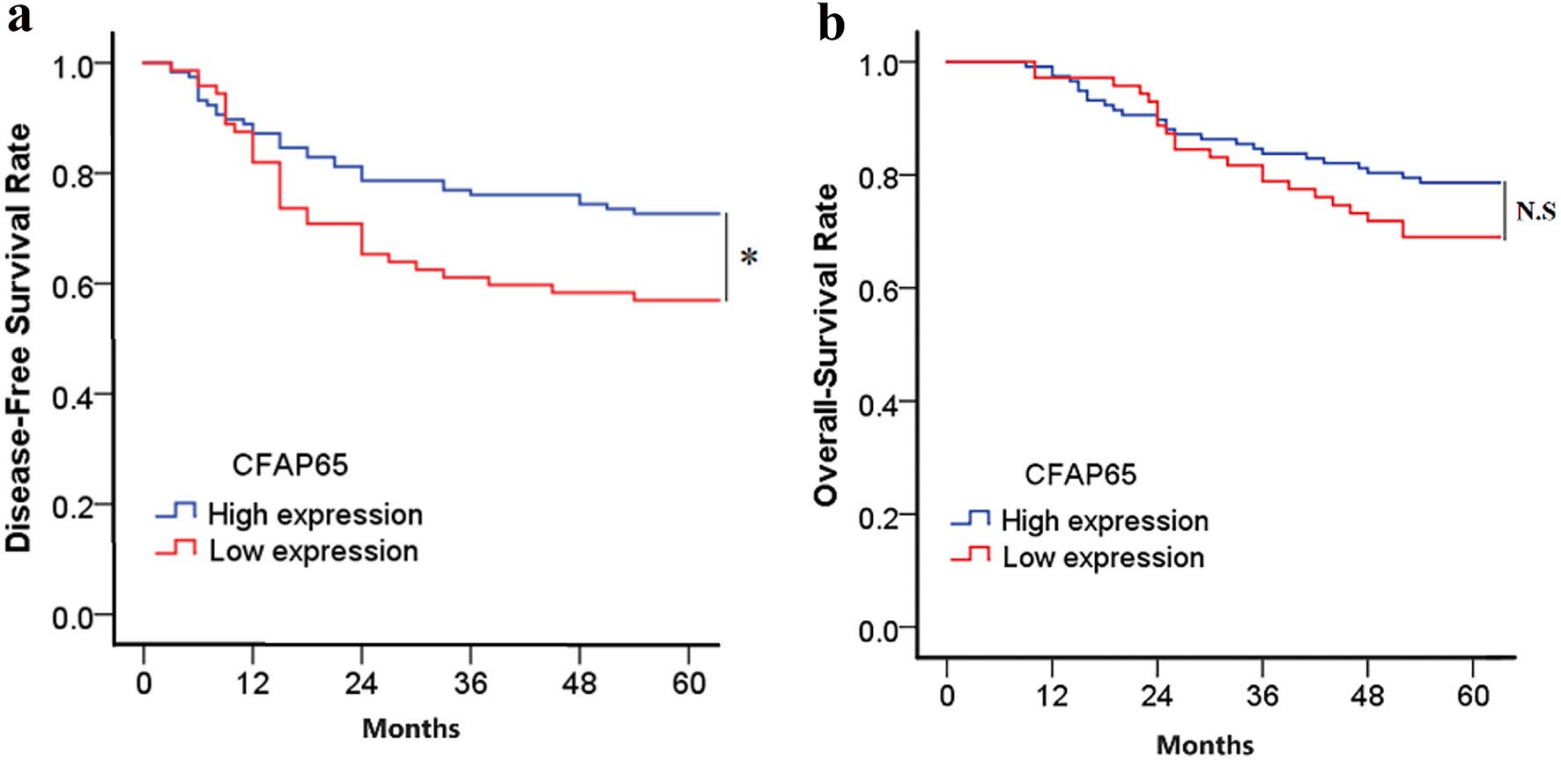
The prognosis of the different expression status of CFAP65. (**a**). The DFS rate; (**b**). The OS rate; *: *P* < 0.05; N.S: No significance

The 5-year DFS and OS rates of all the patients were 66.7% and 75% respectively, with 63 progression and 47 deaths within 5 years after surgery. What is inconsistent with the results in the TCGA database regarding the impact of CFAP65 on the prognosis of colon cancer is that our results indicate there was a significant difference in 5-year DFS between the CFAP65 high- and the low-expression groups (72.6% vs. 56.9%, *P* = 0.03) (Fig. 6a), suggesting high expression of CFAP65 predicts better prognosis, and the 5-years OS rate has no significant difference between high- and low-expression groups (78.6% vs. 69%, *P* = 0.171) (Fig. 6b).

Cox proportional hazard regression model was performed to analyze the independent prognostic factors affecting 5-year DFS rate, including age, histological differentiation, TNM stage, tumor location, mismatch repair deficiency, vascular invasion, and CFAP65. The result demonstrated that CFAP65, TNM stage and tumor location were independent prognostic factors for disease-free survival (Table 3). We designed the receiver operating characteristic (ROC) curve to designate CFAP65 as a prognostic biomarker (Supplementary Fig. 2). The area under curve (AUC) value is 0.616, indicating that the CFAP65 expression has a degree of prognostic value for colon cancer.

**Table 3:** Cox proportional hazard regression model for 5-year DFS

Variable	HR	95% CI of HR	*P* value	
CFAP65	1.694	1.022–2.809		0.041
Age	1.015	0.598–1.723		0.957
Histological differentiation	0.939	0.610–1.446		0.775
TNM stage	2.365	1.414–3.958		0.001
Tumor location	0.704	0.536–0.924		0.012
Mismatch repair deficiency	2.349	0.731–7.547		0.152
Vascular invasion	0.716	0.404–1.270		0.253

## Discussion

Precision medicine in colon cancer therapy faces the dilemma of how to stratify patients based on differential postoperative risk of progression. Established prognostic factors such as TNM stage, lymphovascular invasion, tumor location, and serum CEA are not sufficient to guide individualized adjuvant chemotherapy [16]. With the development of molecular biology, more and more genetic or epigenetic biomarkers are being used in the clinic to assess the risk of colon cancer [17, 18]. Although emerging biomarkers are increasingly improving the accuracy of risk assessment [18, 19], novel biomarkers continue to be needed to support prognosis and treatment decisions due to the wide heterogeneity among colon cancer patients [18].

CFAP65 is thought to play a critical role in the formation of cilia, mutation or dysfunction of which leads to male asthenoteratospermia and sterility [9, 20, 21]. CFAP65 also plays a role in other diseases, for example, as a candidate gene for familial gastroschisis and Parkinson’s disease [22, 23]. In cancer research, some studies suggest that CFAP65 has a tumor suppressive function by promoting cell differentiation and inhibiting tumor progression: Tang et al. reported that CFAP65 promotes neural stem cell differentiation [24], and another study by Lee et al. suggested that CFAP65 inhibits cell proliferation in gastric cancer [10]. However, the biological function and clinical significance of CFAP65 in colon cancer remains unclear. Through GO enrichment analysis, we found that CFAP65-related genes were significantly enriched in pathways related to cell motility and signal transduction in colon cancer, suggesting that CFAP65 may be related to tumor metastasis, which prompted us to further investigate its prognostic significance in the clinic. However, CFAP65 is likely not acting alone. It may bind with other proteins to work together. Based on this speculation, we analyzed the proteins interacting with CFAP65, and found CFAP65 potentially play a role in cell movement and cancer invasion. For example, CFAP65 interacts with CFAP43, CFAP44 and CFAP47 to regulate microtube polymerization or cilium movement, which is associated with cell migration (Supplementary Fig. 3).

Our results showed that low expression of CFAP65 predicted worse DFS. Furthermore, CFAP65 expression is independent of other clinicopathologic variables such as TNM stage and tumor location, suggesting that CFAP65 is an independent biomarker for assessing tumor progression risk. As postoperative recurrence and metastasis remain a major challenge for colon cancer therapy, efficient prognostic markers are helpful to identify the high-risk patients who might benefit from adjuvant chemotherapy [25]. However, this study also has some limitations. First, the phenomenon we found that patients with low expression of CFAP65 have poorer prognosis is limited to clinical samples, and we have not delved into the underlying mechanisms. Then we did not include samples from patients with advanced colon cancer, so the significance of CFAP65 for advanced colon cancer is still unclear. Consequently, we will mainly focus on its molecular mechanism and the significance of CFAP65 in advanced colorectal cancer in the future research. In conclusion, our work contributes to clinical decision making for individualized therapy. On the other hand, the IHC assay for the detection of CFAP65 is a widely used pathological method without technical complexity, suggesting a high translational value of our finding.

## Conclusions

CFAP65 is a potential prognostic marker for colon cancer, contributing to individualized evaluation and therapy for colon cancer patients.

### Electronic supplementary material

Below is the link to the electronic supplementary material.

Supplementary Material 1

Supplementary Material 2

Supplementary Material 3

Supplementary Material 4

Supplementary Material 5

## Data Availability

The data generated in the present study may be requested from the corresponding author.

## Abbreviations

CFAP65: Cilia and Flagella Associated Protein 65
GO: Gene ontology
RT-qPCR: Reverse Transcription and Quantitative Polymerase Chain Reaction
CC: Colon cancer
IHC: Immunohistochemistry
IRS: Immunoreactive score
NC: Nitrocellulose
cDNA: Complementary DNA
DFS: Disease-Free Survival
OS: Overall Survival
MF: Molecular Function
BP: Biological Process
ROC: Receiver Operating Characteristic
AUC: Area Under Curve
