# Supplementary figures and images for: The expression and clinical significance of CFAP65 in colon cancer
Source: BMC Gastroenterol. 2024 Jul 11;24:222. doi: 10.1186/s12876-024-03317-5 (PMC11238475; doi:10.1186/s12876-024-03317-5)

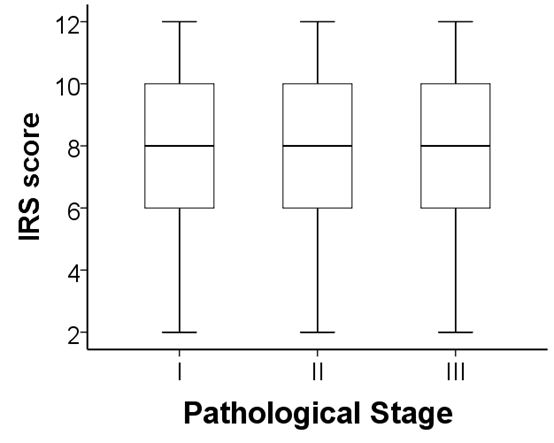

Supplement: Supplementary file 2 — Supplementary Material 2 [file 12876_2024_3317_MOESM2_ESM.tif]

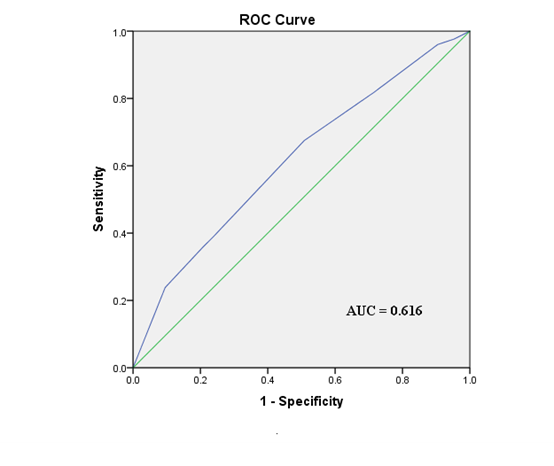

Supplement: Supplementary file 3 — Supplementary Material 3 [file 12876_2024_3317_MOESM3_ESM.tif]

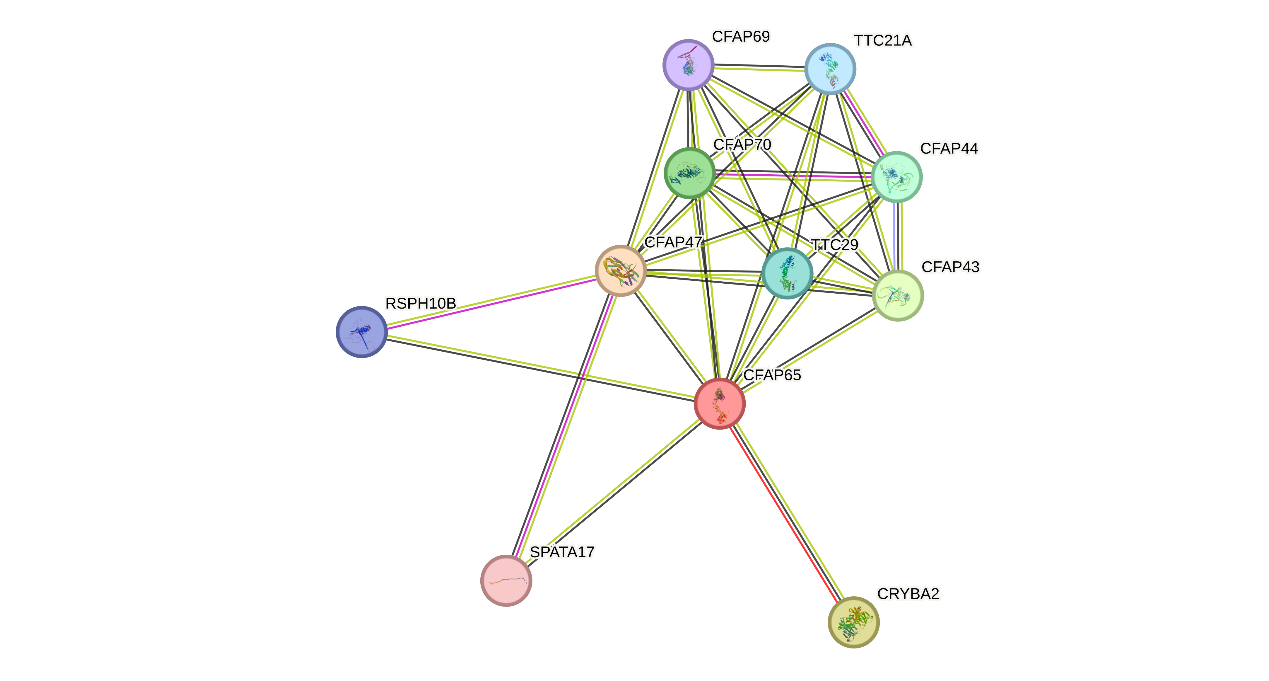

Supplement: Supplementary file 4 — Supplementary Material 4 [file 12876_2024_3317_MOESM4_ESM.tif]

Figure 3

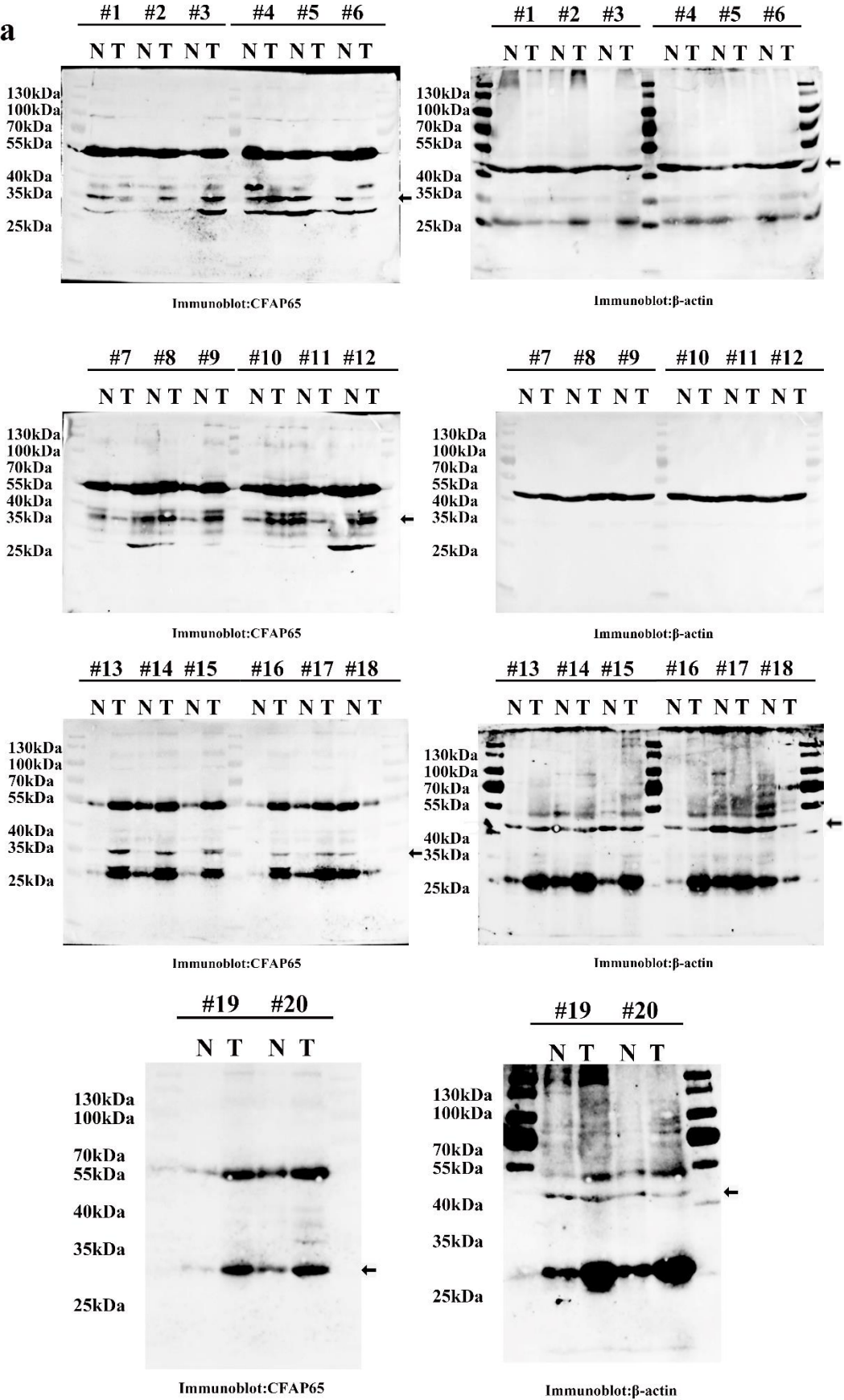

Supplement: Supplementary file 5 — Supplementary Material 5 [file 12876_2024_3317_MOESM5_ESM.pdf]
